# Unequal Benefits: How Parental Education Falls Short for Black and Latino Youth

**DOI:** 10.31586/ojer.2025.1232

**Published:** 2025-02-12

**Authors:** Shervin Assari, Maria Jahromi, Hossein Zare

**Affiliations:** 1 Department of Internal Medicine, Charles R. Drew University of Medicine and Science, Los Angeles, CA, United States; 2 Department of Family Medicine, Charles R. Drew University of Medicine and Science, Los Angeles, CA, United States; 3 Department of Urban Public Health, Charles R. Drew University of Medicine and Science, Los Angeles, CA, United States; 4 Marginalization-Related Diminished Returns (MDRs) Center, Los Angeles, CA, United States; 5 Research School of Economics, Australian National University, Canberra, Australia; 6 School of Economics, University of Sydney, Sydney, Australia; 7 Department of Health Policy and Management, Johns Hopkins Bloomberg School of Public Health, Baltimore, MD, United States; 8 School of Business, University of Maryland Global Campus (UMGC), Adelphi, United States

**Keywords:** School Performance, Achievement Gap, Ethnic Groups, Social Determinants of Health

## Abstract

**Background:**

Parental education is a key determinant of academic performance, yet its protective effects may differ by race and ethnicity. The concept of Minorities’ Diminished Returns (MDRs) highlights the weaker association between socioeconomic resources and outcomes for marginalized populations, including Black and Latino youth.

**Objective:**

To investigate whether the positive association between parental education and school performance (letter grades) is weaker for Black and Latino youth compared to non-Latino White youth.

**Methods:**

Data were drawn from the Monitoring the Future (MTF) 2023 study. The sample included Black, Latino, and non-Latino White youth. The outcome was a nine-level continuous measure of academic performance based on self- reported letter grades, with higher scores indicating better performance. Multivariate regression models tested interactions between parental education and race/ethnicity in predicting grades, adjusting for confounders such as family income, gender, and school characteristics.

**Results:**

A total number of 7584 12^th^ graders entered the study. Parental education was positively associated with school performance across all groups, but the magnitude of this association was significantly smaller for Black and Latino youth compared to non-Latino White youth. Even after controlling for socioeconomic and contextual factors, the racial and ethnic differences in the strength of this association persisted.

**Conclusions:**

Our findings provide evidence of Minorities’ Diminished Returns (MDRs) in the academic domain, with Black and Latino youth experiencing weaker benefits of parental education on school performance. These disparities suggest that structural barriers and systemic inequities undermine the translation of parental educational attainment into academic success for marginalized groups. Policy interventions must address these structural barriers to promote equity in educational outcomes.

## 1. Introduction

Academic performance is a crucial predictor of future success, shaping opportunities in higher education, employment, and economic mobility [1, 2]. For high school students, strong academic achievement serves as a foundational step toward college enrollment, competitive job prospects, and long-term socioeconomic stability [3, 4]. Beyond individual benefits, academic performance also has far-reaching societal implications, influencing workforce quality, innovation, and economic growth [5]. High school students who excel academically are more likely to transition seamlessly into adulthood, equipped with the skills and qualifications needed to navigate an increasingly complex world [6–8].

Parental education is widely recognized as a key driver of children’s academic success [9–11]. Parents with higher educational attainment often possess the knowledge and skills needed to actively support their children’s learning [12]. They are more likely to engage in their children’s academic lives, fostering positive attitudes toward education and encouraging high expectations for performance [13]. Furthermore, higher parental education is associated with greater access to high-quality schools, a broader range of extracurricular opportunities, and better educational resources, including books, technology, and tutoring [14, 15]. Children of highly educated parents are also more likely to develop strong cognitive and behavioral skills, benefiting from enriched home environments and greater exposure to intellectually stimulating activities [13, 16].

In addition to these direct influences, parental education often correlates with higher family income, which can provide additional advantages such as stable housing in safe neighborhoods, access to healthcare, and reduced financial stress [13, 17, 18]. These factors collectively create an environment conducive to academic success [19–21]. However, these benefits are not equitably distributed across racial and ethnic groups [22], as structural barriers and systemic inequities often diminish the protective effects of parental education for marginalized populations.

Emerging research on Minorities’ Diminished Returns (MDRs) [23–30] highlights the limited translation of socioeconomic resources, such as parental education, into improved outcomes for racial and ethnic minority groups. Among Black and Latino youth, the advantages typically associated with higher parental education are often undermined by systemic racism, discriminatory practices, and unequal access to opportunities [31, 32]. For example, even when Black and Latino families achieve higher levels of education, they may face barriers such as underfunded schools, implicit bias in educational settings, and limited access to culturally responsive teaching [33–35]. These challenges weaken the link between parental education and academic performance, perpetuating disparities in educational outcomes.

The present study investigates whether the association between parental education and school performance differs by race and ethnicity among youth in the United States. Using data from the Monitoring the Future (MTF) [36–39] 2023 study, we explore how these relationships vary for Black, Latino, and non-Latino White youth. Our focus is on self-reported letter grades as a measure of academic achievement, with a nine-level continuous scale providing insight into nuanced differences in performance. This research extends the MDRs framework [35, 40–45] to the academic domain, highlighting how structural and systemic inequities weaken the effects of parental education for Black and Latino youth. By identifying these disparities, we aim to inform policies and practices that address barriers to educational equity and promote the well-being of all youth, regardless of race or ethnicity. The findings will contribute to the broader discourse on health and social disparities, emphasizing the need for systemic interventions that go beyond individual-level factors to dismantle the structural barriers that undermine educational equity.

## 2. Methods

### 2.1. Design and Setting

The data for this study were drawn from the Monitoring the Future (MTF) [36–39] 2023 survey, a long-running, nationally representative study that examines trends in behaviors, attitudes, and values among U.S. adolescents. MTF is conducted annually by the University of Michigan’s Institute for Social Research and surveys high school students across the country. Participants in the MTF study are selected using a multistage random sampling procedure to ensure national representativeness. The data collection process includes both in-school and follow-up surveys to capture a wide range of factors influencing adolescent experiences.

For the 2023 wave, data were collected in Spring 2023 and included responses from 12th-grade students (N = 7584 12th graders). This cohort provides a comprehensive snapshot of adolescent behaviors and outcomes during a critical transitional phase, as students prepare to leave high school.

### 2.2. Participants and Procedures

All participants were 12th graders in 2023. Most participants were between 17 and 18 years old, reflecting the typical age range for high school seniors. Data collection involved self-administered questionnaires completed online. MTF’s rigorous sampling and data collection procedures ensure high-quality data that is generalizable to the population of U.S. high school seniors. Participation was voluntary, and respondents were provided with detailed information about the study’s purpose, confidentiality measures, and their right to withdraw at any time without penalty. For this analysis, we only included non- Latino White, Black, or Latino students. Response rate of the 2023 MTF for 12th graders have been 72%.

### 2.3. Ethics

The study was reviewed and approved by the Institutional Review Board (IRB) at the University of Michigan. All procedures were conducted in accordance with the guidelines for the protection of human subjects, ensuring participant safety, privacy, and data confidentiality. Parental consent and participant assent were obtained as appropriate, aligning with ethical standards for research involving minors.

### 2.4. Measures

#### 2.4.1. Outcomes

##### School Performance:

The outcome was a nine-level continuous measure of academic performance based on self-reported letter grades, with higher scores indicating better performance. The specific question read as: “Which one of the following best describes your average grades in this school year?” Responses were 9=“A (93–100)” 8=“A- (90–92)” 7=“B+ (87–89)” 6=“B (83–86)” 5=“B- (80–82)” 4=“C+ (77–79)” 3=“C (73–76)” 2=“C- (70–72)” 1=“D (69 or below)”.

#### 2.4.2. Covariates

Demographic and contextual variables were included as covariates in the analyses to account for potential confounding factors. These variables were measured categorically:

**Age:** Self-reported age was recorded in years.
**City/rural location:** Participants were categorized based on their residence to City/rural location.
**Number of Parents in the Household:** Families were categorized based on the number of parents in the household. This was either zero, one, or two.
**Race:** Participants were categorized as White or Black.
**Ethnicity:** Participants were categorized as non-Hispanic or Hispanic groups.
**Biological Sex at Birth:** Classified as binary (0 = “Female,” 1 = “Male”). **Country Region:** Participants were selected from North-East, Mid-West, South, and West.
**Parental Education:** A continuous variable indicating the highest parental education of the parents from grade schools (1) to graduate studies (6).

These covariates were selected to account for demographic and situational factors that could influence school performance (primary outcome).

### 2.5. Analysis

We began our analysis by examining the descriptive statistics for the pooled sample. Means, standard deviations (SD), and proportions were calculated for all study variables, including parental education (continuous measure), race/ethnicity, and youth outcomes. To explore unadjusted correlations between study variables including parental education, and youth outcomes, we calculated Pearson correlation coefficients for the pooled sample. To test the hypothesis of Minorities’ Diminished Returns (MDRs), we conducted multivariate linear regression analyses using two models. The dependent variable was the youth outcome of interest (e.g., school performance (letter grades)). Independent variables included parental education (continuous measure) and race/ethnicity (categorical variable, with non-Latino White as the reference group). All models controlled for potential confounders, including age, gender, number of parents in the household, geographic region, and other relevant covariates. Model 1 examined the main effects of parental education and race/ethnicity on youth outcomes. Regression coefficients (B), standard errors (SE), 95% confidence intervals (CI), and p-values were reported. This model served as a baseline for understanding the association between parental education and youth outcomes across the pooled sample.

Model 2 included interaction terms between parental education and race/ethnicity (parental education × Black, parental education × Latino) to test whether the relationship between parental education and youth outcomes varied by race/ethnicity. Significant interaction terms (e.g., smaller effect sizes for Black and Latino youth compared to non- Latino White youth) provided evidence for MDRs. Regression coefficients for the interaction terms, along with SEs, 95% CIs, and p-values, were reported.

We used Stata 15 for data analysis, employing survey regression models to account for the complex sampling design. MTF 2023 sample weights were applied to ensure nationally representative estimates. A positive association between parental education and youth outcomes in Model 1 would suggest that higher parental education is generally beneficial across the sample. Negative and significant interaction terms in Model 2 would indicate that the protective effects of parental education are weaker for Black and Latino youth, consistent with the MDRs framework [35, 44, 46–54]. Statistical significance was defined as p < 0.05. Model fit was evaluated using adjusted R-squared values, and residual diagnostics were conducted to ensure the validity of the models.

## 3. Results

### 3.1. Descriptive Data

A total number of 7584 12th graders entered the study. Table 1 provides the descriptive statistics for the pooled sample. Regarding age, 45.6% of the participants were aged 18 years or younger, while 54.4% were older than 18 years (SE = 0.009). In terms of geographic region, the majority of participants (83.8%) resided in a city, while 16.2% lived in a rural area or farm (SE = 0.006). For sex at birth, the sample was nearly evenly split, with 52.9% identifying as female and 47.1% as male (SE = 0.009). Race/ethnicity distribution showed that 52.7% of the participants identified as White, 15.2% as Black, and 32.1% as Hispanic (SEs = 0.009, 0.007, and 0.009, respectively). The mean parental education in the sample was 4.32 (SE = 0.028), and the mean school performance score was 7.09 (SE = .031).

### 3.2. Bivariate Correlations

The bivariate correlations among the study variables in the pooled sample are presented in Table 2. Significant relationships were observed between race/ethnicity and several variables. Black youth were negatively correlated with Hispanic ethnicity (r = - 0.2231, p < 0.05), the presence of two parents in the household (r = −0.2271, p < 0.05), and rural residence (r = −0.0799, p < 0.05), while showing a positive correlation with living in the East (r = 0.0838, p < 0.05) and the South (r = 0.1004, p < 0.05). Hispanic youth showed significant negative correlations with rural residence (r = −0.1768, p < 0.05), parental education (r = −0.2847, p < 0.05), and school performance (r = −0.0963, p < 0.05) but were positively correlated with living in the West (r = 0.2449, p < 0.05). The presence of two parents in the household was positively associated with parental education (r = 0.1521, p < 0.05) and school performance (r = 0.1599, p < 0.05). Age was positively correlated with rural residence (r = 0.048, p < 0.05) but negatively correlated with Hispanic ethnicity (r = - 0.0829, p < 0.05) and living in the West (r = −0.0986, p < 0.05). Regional differences were also observed, with rural residence positively associated with living in the Midwest (r = 0.1723, p < 0.05) and negatively associated with the East (r = −0.0772, p < 0.05), the South (r = −0.0373, p < 0.05), and the West (r = −0.0866, p < 0.05). Parental education was positively associated with school performance (r = 0.2097, p < 0.05) but negatively correlated with Hispanic ethnicity (r = −0.2847, p < 0.05) and Black ethnicity (r = −0.0371, p < 0.05). School performance was negatively correlated with being male (r = −0.1446, p < 0.05) and living in the South (r = −0.061, p < 0.05), while positively associated with living in the Midwest (r = 0.037, p < 0.05).

### 3.3. Multivariable Analysis

Table 3 presents the results of two regression models predicting school performance. The results of Model 1 indicate a strong positive association between parental education and school performance across the pooled sample, with notable disparities by race/ethnicity and geographic region. In Model 2, the significant interaction terms provide evidence for MDRs, highlighting that the protective effects of parental education on school performance are less pronounced for Black and Hispanic youth. These findings underscore the need for policies addressing structural barriers that limit the translation of parental education into educational outcomes for minoritized groups.

Model 1 includes the main effects of demographic variables and parental education, while Model 2 incorporates interaction terms between parental education and race/ethnicity to test for Minorities’ Diminished Returns (MDRs).

#### Model 1: Main Effects

Parental presence was positively associated with school performance (B = 0.323, SE = 0.053, p < 0.001). Parental education was associated with school performance (B = 0.257, SE = 0.023, p < 0.001) indicating that higher parental education and parental presence predict school performance. Age was not significantly associated with school performance (B = −0.101, SE = 0.056, p = 0.071). Male sex was negatively associated with school performance (B = −0.510, SE = 0.055, p < 0.001), indicating lower performance for males compared to females. Black youth (B = −0.610, SE = 0.091, p < 0.001) and Hispanic youth (B = −0.329, SE = 0.076, p < 0.001) had significantly lower school performance compared to non-Hispanic White youth. Residence in the South was associated with lower school performance (B = −0.216, SE = 0.072, p = 0.003), while no significant associations were observed for the Midwest, West, or rural areas.

#### Model 2: Interaction Effects

Parental presence (B = 0.319, SE = 0.053, p < 0.001) and parental education (B = 0.325, SE = 0.033, p < 0.001) remained positively associated with school performance. The interaction between parental education and being Black (B = −0.197, SE = 0.071, p = 0.006) and between parental education and being Hispanic (B = −0.111, SE = 0.051, p = 0.030) were both significant. These results indicate that the positive effects of parental education on school performance are weaker for Black and Hispanic youth compared to their non- Hispanic White peers, consistent with the Minorities’ Diminished Returns (MDRs) framework. After adding interaction terms, the main effects for Black (B = 0.289, SE = 0.336, p = 0.390) and Hispanic youth (B = 0.171, SE = 0.235, p = 0.467) became nonsignificant, suggesting the interaction terms account for differences in school performance by race/ethnicity. Male sex (B = −0.509, SE = 0.055, p < 0.001) and residence in the South (B = - 0.208, SE = 0.072, p = 0.004) remained significant predictors of lower school performance.

## 4. Discussion

The aim of our study was to investigate whether the association between parental education and school performance differs by race and ethnicity among youth in the United States. Specifically, we sought to determine if Black and Latino youth experience diminished returns of parental education on their academic performance compared to non-Latino White youth. Our hypotheses were twofold: first, that parental education would be positively associated with school performance for all groups, and second, that the magnitude of this association would be weaker for Black and Latino youth, reflecting the concept of Minorities’ Diminished Returns (MDRs). The findings of this study confirmed these hypotheses, revealing important insights into the structural barriers that undermine educational equity and highlighting the systemic nature of racial and ethnic disparities in academic outcomes.

Our findings indicate a positive association between parental education and school performance for youth across all racial and ethnic groups. This aligns with established literature emphasizing the role of parental education in fostering academic achievement. Higher parental education typically enables access to resources such as better schools, academic enrichment programs, and extracurricular activities. It also promotes parental engagement, which has been shown to influence students’ attitudes toward learning and overall school performance. However, while this general pattern was evident in our results, the strength of this relationship varied significantly by race and ethnicity, suggesting that structural factors may differentially mediate the benefits of parental education.

For Black youth, the positive impact of parental education on school performance was significantly weaker compared to non-Latino White youth. This finding supports the framework of Minorities’ Diminished Returns (MDRs) [45, 47, 53, 55–63], which posits that socioeconomic resources, such as parental education, yield smaller benefits for racial and ethnic minorities due to systemic barriers. Despite their parents’ higher educational attainment, Black students may face underfunded schools, racial bias within educational institutions, and fewer opportunities for academic support. These structural inequities limit the potential for parental education to translate into optimal academic outcomes, highlighting the persistence of systemic racism in the U.S. education system.

Similarly, Latino youth also exhibited a weaker association between parental education and school performance compared to their non-Latino White peers. This may reflect a combination of structural challenges, such as language barriers, immigration- related stressors, and limited access to culturally responsive educational environments [64–66]. Additionally, many Latino families may reside in areas with under-resourced schools, further diminishing the academic benefits of parental education. These findings extend the MDRs framework to Latino youth, emphasizing the need to address intersecting factors of race, ethnicity, and immigration status that contribute to disparities in educational outcomes.

The interaction effects observed in this study further underscore the complex dynamics of how race, ethnicity, and socioeconomic resources intersect to influence academic performance. While higher parental education is generally expected to provide a protective effect, the significantly weaker impact for Black and Latino youth illustrates the cumulative effects of structural barriers. These interaction effects reflect how racism and discrimination systematically undermine the value of socioeconomic resources for marginalized populations. For non-Latino White youth, the stronger association between parental education and academic performance suggests they are better positioned to capitalize on these advantages, likely due to their greater access to well-resourced schools and supportive academic environments.

Weaker effects of family-level SES indicators on children’s and adolescents’ outcomes can be explained by multiple interrelated mechanisms. Race and ethnicity are highly visible attributes that may lead to discrimination, whereas high SES is less visible and, therefore, less protective for marginalized groups. Consequently, high SES Black and Latino families often face discrimination despite their socioeconomic position, primarily due to the visibility of their racial and ethnic identity. In addition to discrimination, research shows that high SES Black and Latino parents are more likely to be employed in worse jobs compared to their White counterparts, resulting in lower earnings, less wealth accumulation, and greater economic instability. Despite higher SES, these families are more likely to reside in unsafe neighborhoods and send their children to under-resourced schools. Structural inequities such as segregation, social stratification, and the legacy of Jim Crow laws have historically shaped and continue to reinforce these disparities, systematically disadvantaging Black and Latino families compared to White families. Schools attended by Black and Latino children are often less resourced and provide fewer opportunities for academic and extracurricular growth. When Black and Latino students do attend well-resourced schools with a higher percentage of White students, research [67–76] suggests they are disproportionately subjected to discrimination and exclusion, further undermining their potential to benefit from these environments. Racism has also been shown to be negatively associated with general and mental health [76,77], with implications for the children’s wellbeing and their school performance.

These patterns underscore that Minorities’ Diminished Returns (MDRs) are driven by structural, rather than individual, factors [57, 78–86]. Thus, addressing MDRs requires systemic and policy-level interventions rather than focusing solely on families or individuals. Families and students should not be blamed for these disparities, and attributing these findings to inherent differences in IQ is both unfounded and counterproductive. The current social system is structured to confer the greatest benefits to White individuals and families, often at the expense of Black and Latino families. This systemic bias underpins MDRs and demands comprehensive policy reforms to dismantle the structural barriers that perpetuate these inequities.

We conceptualize race and ethnicity as social constructs rather than biological determinants, emphasizing their basis in societal and historical processes rather than inherent genetic differences. The disparities captured by Minorities’ Diminished Returns (MDRs) are not the result of racial determinism or any pseudo-scientific notions of innate inferiority. Instead, MDRs reflect the cumulative impact of structural and historical injustices, such as systemic racism, segregation, and discriminatory policies, which have perpetuated inequities in access to resources and opportunities. These socially constructed categories have been used to stratify society and maintain systems of privilege and disadvantage. It is critical to recognize that MDRs are products of social and historical unfairness, not biological inevitabilities, underscoring the need for structural solutions rather than relying on flawed narratives of racial or ethnic determinism.

### 4.1. Implications

These findings have important implications for educational policy and practice. Addressing the disparities observed in this study requires a focus on structural factors that hinder the translation of parental education into academic success for Black and Latino youth. Policies aimed at equitable funding for schools, the reduction of implicit bias among educators, and the promotion of culturally responsive teaching practices are essential. Moreover, programs that provide targeted academic support for Black and Latino students can help bridge the gap, ensuring that the benefits of parental education are more equitably realized.

### 4.2. Limitations

While this study provides valuable insights into the diminished returns of parental education on school performance for Black and Latino youth, it is important to acknowledge its limitations. One significant limitation is the reliance on self-reported grades as a measure of academic performance. Self-reported data can be subject to recall bias, social desirability bias, or inaccuracies due to misunderstandings of survey questions. As such, the grades reported by students may not fully capture their actual academic achievements, potentially introducing measurement error. Another limitation lies in the scope of the variables included in the Monitoring the Future (MTF) 2023 dataset. While the dataset offers robust information on parental education, school performance, and demographic factors, it does not capture other critical variables that may mediate or moderate the observed associations. For instance, we lacked data on school quality, teacher bias, classroom resources, and peer influences—factors that are known to play a significant role in shaping educational outcomes. Furthermore, the dataset does not account for broader neighborhood characteristics, such as crime rates or access to libraries and educational programs, which could affect school performance. Finally, this study is cross-sectional in design, limiting our ability to infer causal relationships. While we observed significant associations and interactions, the lack of temporal data prevents us from determining the directionality of these relationships or understanding how they evolve over time.

### 4.3. Future Directions

Future research should address these limitations to provide a more nuanced and comprehensive understanding of the diminished returns of parental education on academic performance. First, future studies should utilize objective measures of academic performance, such as standardized test scores or teacher evaluations, to complement self- reported data and reduce potential biases. This would enhance the validity and reliability of the findings. Additionally, expanding the scope of variables examined is crucial. Future studies should include measures of school quality, such as funding levels, teacher qualifications, and student-to-teacher ratios. Exploring how these factors interact with parental education to influence academic performance could shed light on the structural mechanisms driving the observed disparities. Moreover, incorporating neighborhood- level data, such as socioeconomic conditions, access to after-school programs, and community resources, would provide a more holistic view of the environmental contexts shaping educational outcomes. Longitudinal research designs are also needed to examine how the relationship between parental education and school performance evolves over time. Tracking students across multiple years could help identify critical periods when disparities emerge or widen, offering opportunities for targeted interventions. Additionally, such designs could explore how transitions, such as moving to higher- quality schools or neighborhoods, impact the association between parental education and academic success for Black and Latino youth. Finally, qualitative research could complement quantitative findings by exploring the lived experiences of families and students. Understanding how Black and Latino families navigate systemic barriers and how these challenges influence their children’s academic trajectories would provide rich, contextualized insights into the mechanisms underlying Minorities’ Diminished Returns (MDRs) [35, 40–45]. By addressing these gaps, future research can build a more comprehensive evidence base to inform policies and practices aimed at promoting educational equity and dismantling the systemic barriers that perpetuate disparities in academic outcomes.

## 5. Conclusion

In conclusion, our findings highlight significant racial and ethnic disparities in the association between parental education and school performance, emphasizing the enduring impact of structural inequities. The diminished benefits of parental education for enhancing the school performance of Black and Latino youth underscore the urgent need for policies aimed at addressing systemic barriers. These include improving the quality of schooling in under-resourced areas, ensuring equitable distribution of educational resources, and combating discrimination against Black and Latino youth within the education system. In addition to these higher-level policy interventions, efforts to empower parents in these communities to better support their children’s educational journeys are essential. While parental education remains a key determinant of academic success, its benefits are inequitably distributed. Addressing these disparities demands systemic reforms that prioritize equity, ensuring that all youth, regardless of their racial or ethnic background, have the opportunity to reach their full academic potential.

## Figures and Tables

**Table 1.** Descriptive Data

	Proportion (%)	SE
**Age**		
<18 years old	0.456	0.009
≥18 years old	0.544	0.009
**Region**		
City	0.837	0.006
Country/Farm	0.162	0.006
**Region**		
North East	0.173	0.005
Mid-West	0.215	0.005
South	0.371	0.006
West	0.240	0.007
**Sex at Birth**		
Female	0.529	0.009
Male	0.471	0.009
**Race/Ethnicity**		
White	0.527	0.009
Black	0.152	0.007
Hispanic	0.321	0.009
**Parental Presence**		
Zero	0.066	0.004
One	0.291	0.007
Two	0.643	0.007
	Mean	SE
Parental Education	4.317	0.028
School Performance (1 – 9)	7.093	0.031

**Table 2.** Bivariate correlations in the Pooled Sample

	1	2	3	4	5	6	7	8	9	10	11	12
1 Race (Black)	1											
2 Ethnicity (Hispanic)	−0.223*	1										
3 Two Parents Present	−0.227*	−0.014	1									
4 Age (Yr)	0.026	−0.083*	−0.042*	1								
5 Rural/Country	−0.079*	−0.177*	−0.021	0.048*	1							
6 Country Region (East)	0.084*	−0.066*	0.017	−0.026	−0.077*	1						
7 Country Region (Mid-West)	−0.139*	−0.207*	0.045*	0.035*	0.172*	−0.344*	1					
8 Country Region (South)	0.101*	0.108*	−0.062*	0.052*	−0.037*	−0.438*	−0.490*	1				
9 Country Region (West)	−0.074*	0.245*	0.009	−0.099*	−0.087*	−0.188*	−0.210*	−0.267*	1			
10 Sex (Male)	0.026	−0.023	−0.011	0.047*	0.023	0.069*	−0.038*	0.016	−0.067*	1		
11 Parental Education (1–6)	−0.037*	−0.285*	0.152*	0.013	−0.023	0.043*	0.032*	−0.023	−0.071*	0.019	1	
12 School Performance (1–9)	−0.117*	−0.096*	0.160*	−0.019	0.003	0.028	0.037*	−0.061*	0.004	−0.145*	0.210*	1

* p < 0.05 (Pearson)

**Table 3.** Predictors of School Performance

	B	SE	95%	CI	p
**Model 1**					
Age	−0.101	0.056	−0.211	0.009	0.071
Male	−0.510	0.055	−0.618	−0.401	< 0.001
Black	−0.610	0.091	−0.789	−0.432	< 0.001
Hispanic	−0.329	0.076	−0.478	−0.181	< 0.001
Mid West	−0.119	0.075	−0.266	0.029	0.114
South	−0.216	0.072	−0.357	−0.075	0.003
West	−0.165	0.114	−0.389	0.058	0.147
Country / Farm	0.061	0.076	−0.089	0.211	0.425
Parental Presence (1–2)	0.323	0.053	0.219	0.426	< 0.001
Parental Education (1–6)	0.257	0.023	0.211	0.303	< 0.001
Intercept	6.099	0.158	5.789	6.409	< 0.001
**Model 2**					
Age	−0.103	0.056	−0.212	0.007	0.067
Male	−0.509	0.055	−0.617	−0.401	< 0.001
Black	0.289	0.336	−0.370	0.948	0.390
Hispanic	0.171	0.235	−0.289	0.631	0.467
Mid West	−0.114	0.075	−0.262	0.033	0.129
South	−0.208	0.072	−0.349	−0.068	0.004
West	−0.182	0.115	−0.407	0.044	0.114
Country / Farm	0.072	0.077	−0.078	0.223	0.344
Parental Education (1–2)	0.319	0.053	0.215	0.423	< 0.001
Parental Education (1–6)	0.325	0.033	0.260	0.391	< 0.001
Parental Education x Black	−0.197	0.071	−0.337	−0.057	0.006
Parental Education x Hispanic	−0.111	0.051	−0.211	−0.011	0.030
Intercept	5.770	0.194	5.389	6.151	< 0.001

## Data Availability

MTF data are available to public at University of Michigan ICPSR: Monitoring the Future: A Continuing Study of American Youth (12th-Grade Survey), 2023 https://www.icpsr.umich.edu/web/NAHDAP/studies/39172
